# 磷脂酰丝氨酸分子印迹聚合物对血浆外泌体的富集与蛋白质组学分析

**DOI:** 10.3724/SP.J.1123.2024.05003

**Published:** 2025-05-08

**Authors:** Xianhui CHENG, Wenjing YU, Dongxue WANG, Liyan JIANG, Lianghai HU

**Affiliations:** 1.吉林大学生命科学学院, 吉林 长春 130023; 1. School of Life Sciences, Jilin University, Changchun 130023, China; 2.慧眼大科学设施, 智慧医学国际研究院, 广东 广州 510000; 2. The π-HuB Project Infrastructure, International Academy of Phronesis Medicine, Guangzhou 510000, China

**Keywords:** 液相色谱-串联质谱, 外泌体, 血浆, 蛋白质组学, 胰腺癌, 分子印迹, 磷脂, liquid chromatography-tandem mass spectrometry (LC-MS/MS), exosome, plasma, proteomics, pancreatic cancer, molecular imprinting, phospholipids

## Abstract

外泌体是肿瘤标志物的重要来源,血浆作为最常用的临床体液之一,其组成较为复杂且存在大量高丰度蛋白质的干扰,如何有效地从血浆中分离外泌体是临床研究的重要挑战之一。本研究将磷脂酰丝氨酸分子印迹聚合物(PS-MIP)用于血浆外泌体的富集,PS-MIP能够特异性识别外泌体质膜上的磷脂酰丝氨酸,从而实现外泌体的高选择性富集。该方法被用于3例健康志愿者和3例胰腺癌患者的血浆蛋白质组学分析和潜在肿瘤标志物的筛选,在健康对照组中的血浆外泌体中鉴定到了1052种蛋白质和4545条肽段,胰腺癌患者血浆外泌体中鉴定到了972种蛋白质和4096条肽段。将蛋白质组学鉴定到的外泌体蛋白质与包含所有细胞外囊泡分子信息的Vesiclepedia数据库进行比较,结果表明84%的PS-MIP富集的血浆外泌体蛋白质存在于该数据库;与只包含外泌体分子信息的ExoCarta数据库进行比较,发现PS-MIP法鉴定出了ExoCarta数据库中Top 100外泌体蛋白质中的77种。与健康对照组相比,胰腺癌患者血浆外泌体中表达量上调的蛋白质有11个,下调的蛋白质有24个。蛋白相互作用网络(PPI)分析显示,相关性较高的前3个蛋白质是补体因子D(CFD)、补体C3(C3)和血管性血友病因子(VWF),在胰腺癌患者外泌体的蛋白质组学表达上调的蛋白质中,外泌体蛋白样糖基转移酶2(EXTL2)、*α*-2-巨球蛋白样1(A2ML1)和人帕金森病蛋白7(PARK7)的差异最为显著,这些蛋白质可能是胰腺癌诊断和预后评估的潜在生物标志物,为胰腺癌的早期诊断和预后提供了重要的科学依据。

## Abstract (en)

Exosomes are 40-160 nm vesicular nano-bodies secreted by most cells that carry large amounts of biologically active substances originating from the parent cell. Proteins in exosomes are protected by phospholipid bilayer membranes that protect them from degradation by enzymes within body fluids. Along with nucleic acid, proteins and metabolites, exosomes are biomolecules that are considered to be among the most important for discovering tumor markers. Plasma is among the most commonly used body fluids in clinical settings; it is highly complex and contains many proteins and metabolites that interfere with exosome isolation. Consequently, the development of methods for effectively isolating exosomes is a key challenge prior to their use in clinical research.

In this study, we used a phosphatidylserine molecularly imprinted polymer (PS-MIP) to enrich plasma exosomes. Subsequent immunoblotting analyses for the CD9, TSG101, and CD81 exosome marker proteins showed that signals can be detected using only 5 μL of plasma, thereby demonstrating the efficiency and specificity of the enrichment protocol. Transmission electron microscope (TEM) and nanoparticle tracking analysis (NTA) data revealed that the enriched vesicles are 30-100 nm in size with elliptical or cup-shaped structures, consistent with the morphology and particle-size-distribution characteristics of the exosomes, suggesting that PS-MIP is capable of successfully isolating exosomes. Nanoflow cytometry revealed that 75.4% of the multi-angle laser scattering (MALS) signal is derived from the PS-MIP-enriched exosomes, which indicates that these enriched exosomes are highly pure and free of interference from impurities, such as aggregated protein particles that are similar in size to the exosomes themselves. This method was used to analyze the proteomes and potential exosomal protein markers of clinical plasma samples from three pancreatic-cancer patients and three healthy volunteers. A total of 1052 proteins and 4545 peptides were identified in the plasma exosomes of healthy volunteers, with a total of 972 proteins and 4096 peptides identified in the plasma exosomes of the pancreatic-cancer patients. Further bioinformatics analyses revealed that the Vesiclepedia database covered 84% of the proteins identified in the plasma exosomes isolated using the PS-MIP method; these proteins comprise 77 of the 100 most frequently identified exosomal proteins in the ExoCarta database. The identified proteins from the cellular components were subjected to gene ontology (GO) analysis, which revealed that they are mainly derived from the exosomes, thereby demonstrating the high selectivity of the PS-MIP method for enriching plasma exosomes and providing specificity for subsequent tumor-marker screening. Label-free quantitative analysis showed that 11 proteins were upregulated and 24 proteins were downregulated in the plasma exosomes of patients with pancreatic cancer compared to those of healthy volunteers. The highly expressed and lowly expressed proteins in the plasma exosomes of patients with pancreatic cancer were subjected to GO, which showed that highly expressed proteins related to the positive regulation of metabolic and biological processes were found in the plasma exosomes of patients with pancreatic cancer compared to those of healthy volunteers, whereas the most significantly under-expressed proteins are related immune-system processes, followed by stimulus-responsive, multicellular bioprocesses, bioregulatory, and interspecies-interacting biological-process-related proteins. The top three proteins, which are relatively highly correlated through protein-protein interaction networks (PPI) analysis, were determined to be complement factor D (CFD), complement component 3 (C3), and von Willebrand factor (VWF). Among the upregulated proteins in the exosomes of patients with pancreatic cancer, exostosin-like glycosyltransferase 2 (EXTL2), *α*-2-macroglobulin like 1 (A2ML1), and Parkinson’s disease protein 7 (PARK7) were the most significantly overexpressed. Hence, these proteins are potential biomarkers for the diagnostic and prognostic assessment of pancreatic cancer and may provide support for further clinical studies into pancreatic cancer.

外泌体是由细胞分泌的具有磷脂双分子层的细胞外囊泡,直径范围为40~160 nm, 1981年Trams等人将源自质膜的囊泡统称为“exosome”,曾被认为是细胞分泌的废弃物^[1]^。深入研究发现,外泌体不仅介导细胞间的信息传递作用,而且在维持器官稳态和疾病分子机制研究中发挥着重要作用^[2]^。外泌体携带了源自母细胞的大量蛋白质、核酸、代谢物和脂质等,与疾病发生发展有着密切的联系^[3]^,由于受磷脂双分子层膜的保护,可免受蛋白酶的降解,因此外泌体是生物标志物的重要来源^[4]^。

血液循环保证着机体新陈代谢的正常进行,不同组织分泌的外泌体也会进入循环系统,因此,血浆是肿瘤标志物筛选的重要来源之一^[5]^。由于血浆组成复杂,高丰度蛋白质含量极高,如何将外泌体进行分离富集,仍具有较大的挑战性。目前,超速离心方法被认为是外泌体分离的“金标准”^[6]^,但超速离心存在回收率和通量较低、依赖昂贵的仪器等不足,其他方法如聚合物沉淀法、尺寸排阻色谱法、免疫亲和法等也被用于外泌体的分离^[7]^,但这些方法仍在富集效率、灵敏度和分离通量等方面存在较大的局限性。我们利用反相微乳液体系制备了磷脂酰丝氨酸分子印迹聚合物(PS-MIP)^[8]^,通过与暴露在外泌体膜外小叶中的PS相互作用,从而实现外泌体的亲和富集。与超速离心方法相比,该法具有较高的富集效率,且该材料具有超顺磁性,可与自动化提取仪偶联实现样品的高通量处理,为临床大队列样品的分析提供了有力的手段。

胰腺癌被称为“癌症之王”^[9]^,由于胰腺组织隐匿在肝脏下沿,神经系统不发达,早期没有明显的症状,其较高的死亡率和较短的病程给传统的化疗带来了巨大挑战^[10]^,基因组学研究仍然无法确定其早期检测和治疗的靶点^[11]^,糖类抗原19-9(CA 19-9)是目前临床常用的胰腺癌诊断和预后标志物^[12]^,但由于其敏感性和特异性较低,难以实现早期的筛查。蛋白质组作为基因组的重要补充和功能体现,能够为胰腺癌的早期诊断、靶向治疗和预后监测提供更好的标志物来源^[13]^。

本研究中我们制备了PS-MIP并将其应用于血浆外泌体的富集和胰腺癌患者样本的蛋白质组学分析。PS-MIP表面具有PS印迹空腔,可以与外泌体质膜上的PS特异性结合,所富集的外泌体经免疫印迹、透射电子显微镜(transmission electron microscope, TEM)、纳米粒子追踪分析(nanoparticle tracking analysis, NTA)和流式细胞术等分析以验证其富集的效率和完整性;对胰腺癌患者和健康对照者的临床样本进行了无标记的定量蛋白质组学分析,对得到的结果进行生物信息学分析,筛选到了若干潜在的胰腺癌生物标志物,为肿瘤的液体活检提供了新的手段和方法。

## 1 实验部分

### 1.1 仪器、试剂与材料

Talos L120C透射电子显微镜(美国Thermo Scientific); RF-6000荧光分光光度计(日本岛津); CentriVap真空离心浓缩仪(美国Labconco); Nanosight NS300纳米粒子跟踪分析仪(英国马尔文帕纳科); nanoElute 2纳升级超高效液相色谱(德国Bruker Daltonics); timsTOF Pro 2捕集离子淌度质谱(德国Bruker Daltonics);高分辨率纳米流式细胞仪(英国A50 micro plus Apogee);Image Quant LAS 4000mini (美国GE Healthcare)。

3 -脲丙基三乙氧基硅烷(UPTES,40.0%~50.0%,甲醇溶液)、四乙氧基硅烷(TEOS,98%)、PS(97%)、甲苯(分析级)、乙腈(色谱级)、甲酸(色谱级)和0.25%胰蛋白酶来自Sigma公司(美国密苏里州圣路易斯市);甲醇(色谱级)、乙醇(分析级)、盐酸(分析级)和氨水(28%)购自国药集团化学试剂有限公司(上海);四水氯化亚铁(99%)和六水氯化亚铁(97%)购自阿拉丁工业公司(上海); *N*-十六烷基三甲基溴化铵(CTAB,分析级)和丙酮(分析级)购自J& K Scientific Ltd.(上海);透析膜(3500 Da)购自Solarbio公司;磷酸缓冲液(PBS)购自普诺赛生命科技有限公司 (武汉);RIPA裂解液购自Thermo Fisher Scientific(美国);BCA蛋白浓度检测试剂盒购自碧云天生物技术有限公司(上海);CD63(兔源,1∶3000, ab134045)、TSG101特异性抗体(鼠源,1∶3000, ab83)购自Abcam Plc(美国);CD9特异性抗体(兔源,1∶3000,D3H4P )购自Cell Signaling Technology(美国);化学发光辣根过氧化物酶(HRP)底物(ECL)WB显影液购自Merck Millipore(德国);CD81(兔源)特异性抗体、HRP-Anti-Rabbit和HRP-Anti-Mouse二抗购自Santa cruz(美国)。

### 1.2 样本采集

研究经吉林大学第二医院医学伦理委员会批准,批准号为SB-2023-008。参与者均在清晨空腹状态下采集全血,经抗凝血和离心处理后得到血浆原始样本,于-80 ℃冻存。健康志愿者年龄在18岁以上,无恶性肿瘤、免疫缺陷、自身免疫性疾病、肝炎或人类免疫缺陷病毒(HIV)感染史。血浆解冻后以2500 g的转速离心15 min,去除细胞碎片,收集上清液作为预处理的血浆样本。

### 1.3 PS-MIP的制备

PS-MIP根据我们之前报道的方法^[8]^合成,简述如下:首先将0.020 mol CTAB分散在100.0 g干燥的甲苯油相中,在氮气条件下,加入含1.724 mmol FeCl_2_·4H_2_O和3.448 mmol FeCl_3_·6H_2_O的9.44 mL水溶液,剧烈搅拌1 h,缓慢加入1 mL氨水(28%水溶液),整个体系立即变黑,表明形成了Fe_3_O_4_纳米复合材料;然后加入20 μmol PS作为模板分子,充分搅拌20 min,滴加60 μmol的UPTES和TEOS混合物(物质的量比为0.5∶9.5),并在室温氮气环境下机械搅拌6 h。再加入20 mL丙酮使乳液破裂,将1.5 mL盐酸(37.2%)与150 mL甲醇混合,形成甲醇酸溶液作为萃取溶剂,使用透析膜(截留分子质量为3.5 kDa)进行索氏提取24 h,去除表面活性剂和印迹模板,最后在60 ℃真空条件下干燥,即得到PS分子印迹聚合物。

### 1.4 PS-MIP富集血浆外泌体

将5 mg PS-MIP置于0.5 mL PBS 中超声20 min,然后加入预处理的血浆,在4 ℃冷藏室旋转振荡器上培养30 min,磁吸收集富集了外泌体的PS-MIP,加入500 μL PBS轻微振荡洗涤3次,并弃去废液,加入200 μL 0.2 mmol/L氨水溶液,剧烈振荡10 min,洗脱PS-MIP富集的外泌体,最后磁吸收集PS-MIP以备再次使用,收集的外泌体洗脱液经冷冻干燥后保存在-80 ℃。

### 1.5 免疫印迹分析

首先,将5 mg PS-MIP加入5 μL血浆中,富集得到外泌体,然后将RIPA裂解液加入该外泌体中,冰上裂解30 min,使用BCA试剂盒(P0010S, Beyotime)测定裂解后的混合溶液蛋白质浓度,控制蛋白质浓度为1.5~2 μg/μL,样品量为20 μL,用于蛋白质免疫印迹分析。外泌体标记蛋白CD9、CD63、TSG101和CD81(1∶1000, sc-166029, Santa)被用作一抗,并用HRP连接的相应二抗(sc-2357, Santa Crus Biotechnology)进行信号放大,使用Luminata Western化学发光HRP底物(Merck Millipore)和Image Quant LAS 4000mini(GE Healthcare)记录Western印迹信号。

### 1.6 透射电子显微镜检测

将PS-MIP富集的外泌体重新分散在10 μL PBS中,将外泌体液滴点在涂有碳膜的铜网上,并在室温下湿润1 min,用滤纸吸去边缘多余的液体后,加入5 μL 2%磷钨酸(pH值6.5~7.0),对铜网进行负染色1 min,用蒸馏水清洗铜网并在黑暗环境中风干,最后在120 kV的加速电压下使用透射电子显微镜对涂有外泌体的铜栅进行检测。

### 1.7 纳米粒子追踪分析

外泌体粒径和密度分布分析采用纳米粒子跟踪分析仪进行,该仪器配备了激光光源和高灵敏度摄像机,实验设置严格控制如下:摄像头级别为15,温度设置为25.0 ℃,黏度调整为0.86 cP,每秒帧数设置为25,测量时间为60 s,检测阈值为8,在分析之前,用PBS适当稀释样品,以确保最佳测量浓度,使用NanoSight NTA 3.4版软件对获取的数据进行细致分析。

### 1.8 蛋白质组学分析

将富集的外泌体进行裂解得到蛋白质组分,用BCA试剂盒测定浓度后,按照蛋白质与胰蛋白酶的质量比为50∶1在37 ℃酶解16 h,经过C18固相萃取柱纯化后,进行液相色谱-串联质谱分析。采用ReproSil-Pur C18-AQ液相色谱柱(100 μm×30 cm, 1.9 μm),柱温设定为55 ℃,流动相A和B分别为含0.1%(v/v)甲酸的水和含 0.1%(v/v)甲酸的80%(v/v)乙腈水溶液,梯度时间是75 min,流速为300 nL/min,质谱数据采集采用数据依赖扫描(DDA)程序,在同步累积连续碎裂(PASEF)模式下进行,共进行4次PASEF MS/MS扫描,采集的数据通过MSFragger进行数据库检索^[14]^。

## 2 结果与讨论

外泌体表面存在大量的PS,虽然可以利用PS结合蛋白质(如Tim4蛋白^[15]^等)来分离外泌体,但配体蛋白质价格昂贵且储存耐久性较差,PS-MIP作为化学合成的人工抗体,具有成本低和稳定性好的特点。PS-MIP基于亲和力识别外泌体质膜外叶的PS,可从复杂体液中快速分离完整的外泌体。本研究总体流程如图1所示,首先进行PS-MIP外泌体富集效率的评价,然后对胰腺癌患者血浆外泌体进行高通量的LC-MS/MS蛋白质组学研究,以筛选胰腺癌的潜在蛋白质标志物。

**图1:**
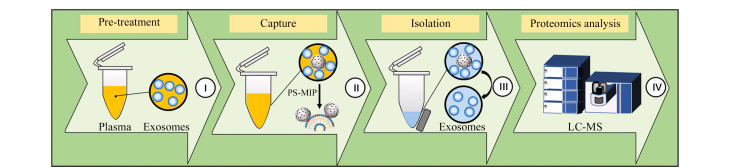
PS-MIP富集血浆中的外泌体用于蛋白质组学研究

### 2.1 PS-MIP富集外泌体的表征

蛋白质免疫印迹是外泌体定性检测的常规手段,CD81、CD63、CD9和TSG101是最常用的4种外泌体标志蛋白,它们参与外泌体的形成和分泌过程。为了评估血浆样本的用量对外泌体富集效果的影响,用5 mg PS-MIP与不同体积的血浆(5、10、20、50、100 μL)振荡孵育30 min,并检测外泌体标记蛋白TSG101、CD81、CD63、CD9的荧光信号。结果表明,仅用5 μL血浆即可检测到外泌体标志蛋白的信号(图2a),考虑到血浆中含有的高丰度蛋白质与代谢物等杂质会抑制PS-MIP与外泌体的吸附,因此采用可获得免疫荧光信号且受干扰最小的5 μL血浆进行外泌体的富集。

**图2:**
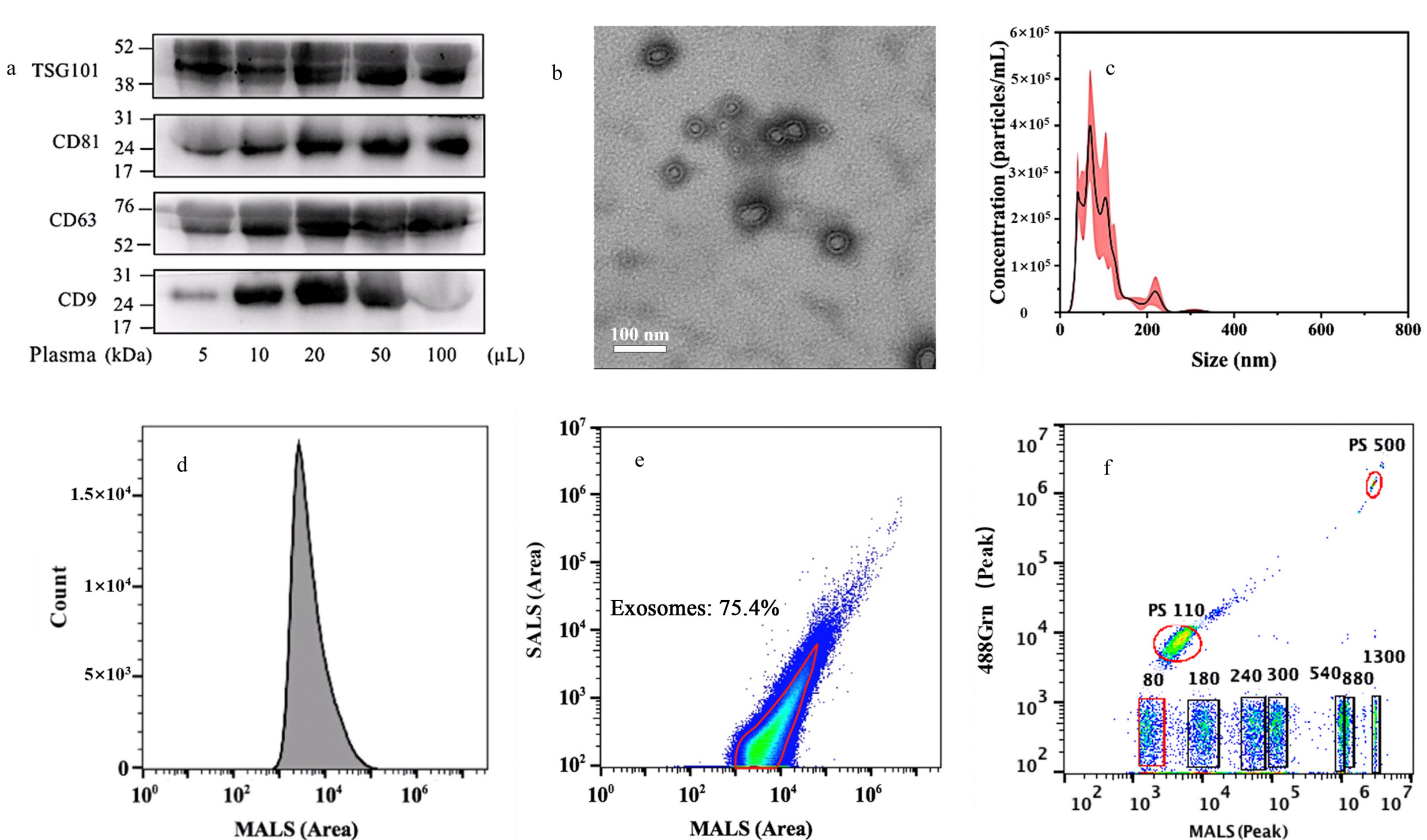
PS-MIP所富集外泌体的相关表征

TEM结果如图2b显示,PS-MIP富集的血浆外泌体,其大小在30~100 nm范围内,呈椭圆形或杯状囊泡结构。NTA结果如图2c显示,PS-MIP富集的外泌体的水合粒径分布较窄,数据以平均值±标准差表示(*p*>0.05),血浆中外泌体的平均粒径为(81.5±7.3) nm, 90%的颗粒数的尺寸值为(142.4±21.6) nm,并测得血浆外泌体含量为(2.66±0.11)×10^7^ particles/mL。高分辨率纳米流式细胞仪结果如图2d和图2f所示,PS-MIP富集的外泌体的水合粒径分布主要集中在80~180 nm范围内,且多角度激光散射(MALS)强度分布较为集中(图2d),纳米流式细胞术结果中75.4%的MALS信号来自PS-MIP富集的外泌体(图2e),这表明PS-MIP富集的外泌体纯度很高,可以排除与外泌体大小相似的蛋白质聚集颗粒等杂质的干扰,粒径数据均是通过与标准微珠(488Grn-MALS)的纳米流式结果(如图2f )比较得出。TEM和NTA结果证明PS-MIP成功分离了外泌体,且符合外泌体的形貌和粒径分布特征。

### 2.2 血浆外泌体的蛋白质组学分析

为研究胰腺癌患者与健康志愿者组间外泌体蛋白质组的差异,我们采用非标记定量的方法分析了3名胰腺癌患者和3名健康志愿者血浆中的外泌体。结果图3a所示,3例健康志愿者(N1~N3)血浆外泌体中平均鉴定出1052种蛋白质和4545条肽段,3例胰腺癌患者(C1~C3)血浆外泌体中平均鉴定出972种蛋白质和4096条肽段,患者血浆外泌体样本中鉴定出的蛋白质和肽的数量和健康志愿者差别不大。进一步定量分析发现胰腺癌患者和健康志愿者血浆外泌体的组内样本的皮尔逊相关系数分别高于0.990和0.980,而组间样本的皮尔逊相关系数则低于0.940,表明质谱结果中组内样本蛋白质表达差异较小,而组间样本蛋白质表达差异较大(图3b)。

**图3:**
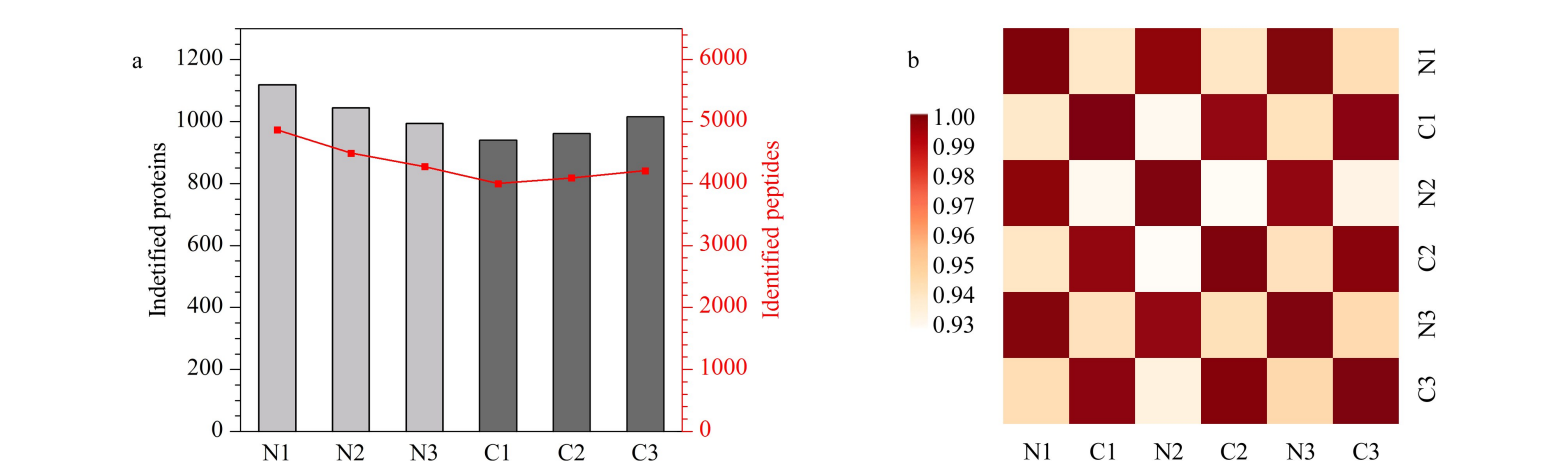
胰腺癌患者(C1~C3)与健康志愿者(N1~N3)的血浆外泌体蛋白质组学分析

将蛋白质组学鉴定到的外泌体蛋白质与包含所有细胞外囊泡分子信息的Vesiclepedia数据库进行比较,结果表明84%的PS-MIP富集的血浆外泌体蛋白存在于该数据库中;与只包含外泌体分子信息的ExoCarta数据库进行比较,发现PS-MIP法鉴定出了ExoCarta数据库中Top 100外泌体蛋白质中的77种(图4a)。对鉴定出的蛋白质进行了细胞成分的基因本体(GO)分析,结果表明鉴定出的蛋白质主要来自外泌体(图4b)。上述结果表明了PS-MIP对血浆外泌体富集的高选择性,为后面肿瘤标志物筛选的特异性提供了保障。

**图4:**
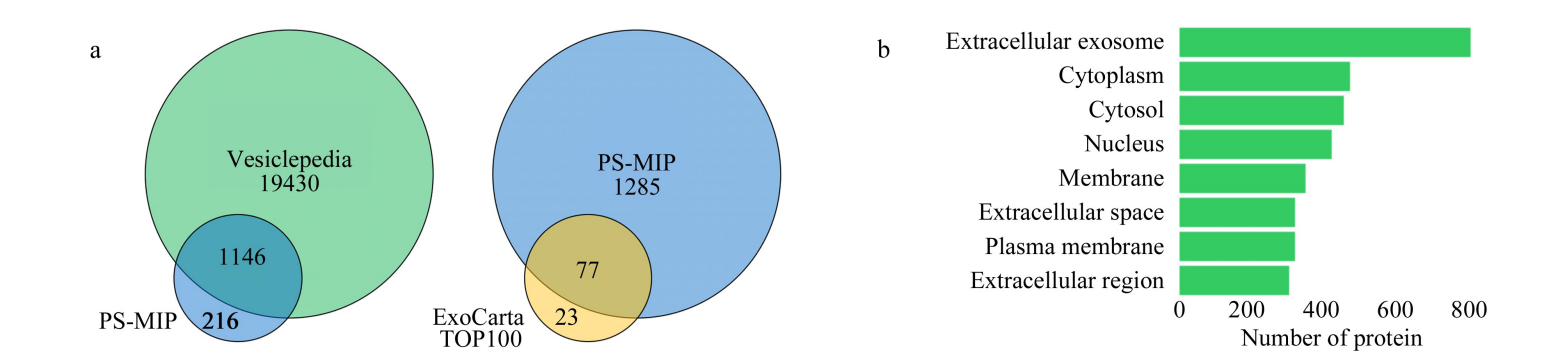
(a)PS-MIP富集的血浆外泌体的蛋白质与Vesiclepedia数据库和ExoCarta数据库的比较及其(b)细胞组分的GO分析

### 2.3 胰腺癌患者与健康志愿者血浆外泌体差异表达分析

胰腺癌患者和健康志愿者的血浆外泌体中具有明显丰度差异的蛋白质火山图分析如图5a所示,结果表明胰腺癌患者血浆外泌体中有11个蛋白质表达量上调,有24个蛋白质表达量下调,这些具有显著性差异的35个蛋白质需要进行系统的生物信息分析,且表达量上调的11个蛋白质中的原肌球蛋白4(tropomyosin,TPM4)、造血细胞特异性Lyn底物(hematopoietic cell-specific Lyn substrate,HCLS)、*α*-2-巨球蛋白样蛋白(*α*-2-macroglobulin like 1,A2ML1)、帕金森病相关蛋白7(Parkinson’s disease-associated protein 7,PARK7)、酰基辅酶1胆固醇酰基转移酶(acyl-CoA cholesterol acyltransferase,ACAT1)、黏附调节分子1(adhesion regulator molecule 1,ADRM1)和血清淀粉样蛋白A4 (serum amyloid A4,SAA4)曾被报道与胰腺癌生物学特性相关。本研究对胰腺癌患者血浆外泌体中的高表达和低表达蛋白质进行了GO分析(图5b),结果表明,与健康志愿者相比,胰腺癌患者血浆外泌体中高表达的是代谢过程和生物过程的正向调节相关蛋白质,而低表达最显著的是免疫系统过程相关蛋白质,其次是刺激响应、多细胞生物过程、生物调节、生物种间相互作用的生物过程相关蛋白质。

**图5:**
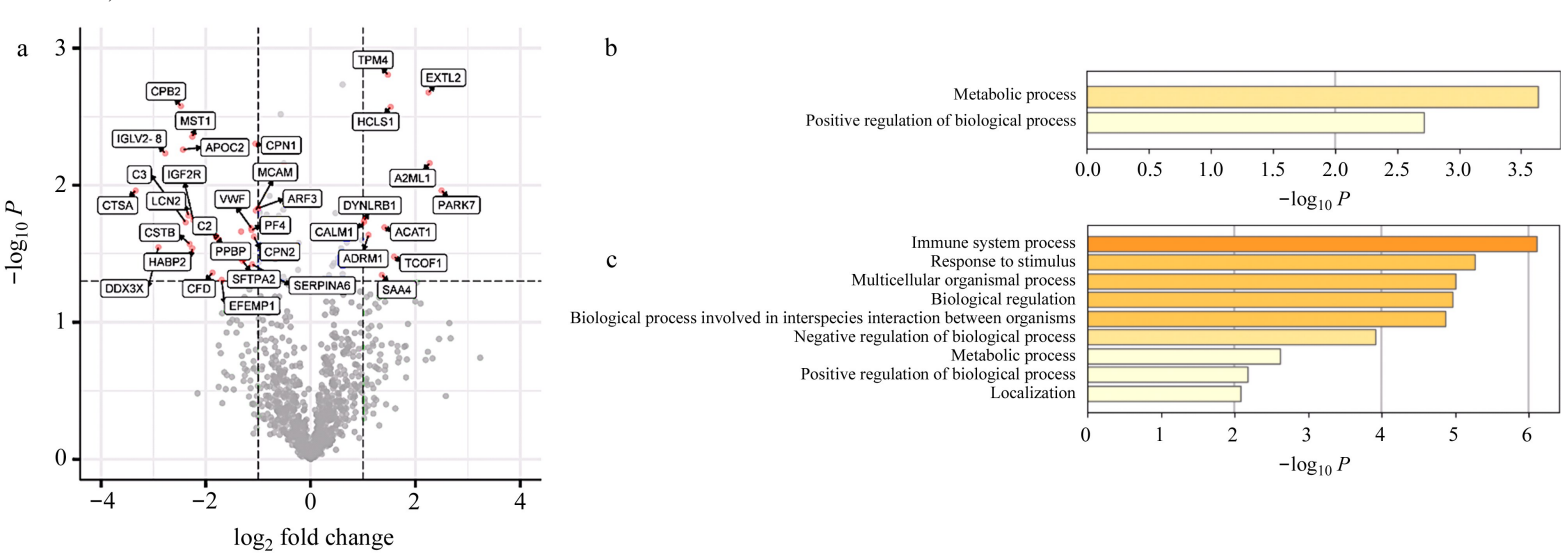
胰腺癌患者与健康志愿者血浆外泌体差异表达蛋白质分析

进一步探讨胰腺癌患者血浆外泌体生物标志物,发现外泌体蛋白样糖基转移酶2(exostosin like glycosyltransferase 2,EXTL2)、造血细胞特异性Lyn底物1(hematopoietic cell-specific Lyn substrate 1,HCLS1)、A2ML1、动力蛋白轻链路障型 1(dynein light chain roadblock-type 1,DYNLRB1)、PARK7、磷酸化酶激酶1(phosphorylase kinase 1,CALM1)、ACAT1、ADRM1、细胞质核糖体生物发生因子1(treacle ribosome biogenesis factor 1,TCOF1)和SAA4在胰腺癌患者血浆外泌体的蛋白质组学结果中表达上调,其中EXTL2、A2ML1和PARK7的过度表达最为显著。EXTL2是硫酸肝素生物合成过程中的一个重要糖基转移酶,它负责在新生硫酸肝素链上交替添加*β*-1-4连接的葡萄糖醛酸(GlcA)和*α*-1-4连接的*N*-乙酰葡糖胺(GlcNAc)单元,而硫酸肝酯又在细胞相互作用、信号传导和发育中发挥着关键作用,EXTL2参与肿瘤细胞生长、迁移、侵袭调控、预后恶化以及胚胎发育和骨骼形成等生理过程,EXTL2的异常表达与多种肿瘤的发生和发展相关,包括乳腺癌、结直肠癌、肝癌和胃癌等^[16]^。*A2ML1*基因可预测肺癌的预后,其表达与TRIM58/cg26157385甲基化相关^[17]^, A2ML1也可作为胰腺癌治疗、诊断和预后的潜在新生物标志物^[18]^。PARK7(DJ-1)通过激活SRC/细胞外信号调节激酶(ERK)/uPA促进胰腺癌细胞的侵袭和转移^[19]^,当PARK7与转录因子TFII-I协同作用时,应对各种损伤并维持胰腺β细胞的功能^[20]^。*TPM4*基因的高表达与胰腺癌患者肿瘤浸润免疫细胞正相关,功能富集分析表明,它可能参与细胞黏附并促进肿瘤细胞迁移^[21]^。*HCLS*基因的表达在慢性淋巴细胞白血病(CLL)的淋巴细胞迁移和归巢调控中起着核心作用,并影响着组织的侵袭和浸润^[22]^,是胰腺癌患者重要的预后生物标志物^[23]^。胆固醇酯(CE)在人胰腺癌标本和细胞系中异常积累是由ACAT-1所介导^[24]^,在胰腺癌和前列腺癌中,ACAT-1介导的CE积累与患者存活率低呈正相关^[25]^。*ADRM1*作为胰腺癌淋巴转移相关基因,在高度淋巴转移的人类胰腺癌细胞系BxPC-3-LN中上调^[26]^。SAA4是检测和监测胰腺癌进展性生长的候选生物标志物^[27]^。DYNLRB1蛋白被认为是特定载体所需的辅助亚基,对一般的动力蛋白介导的运输和感觉神经元的存活至关重要^[28]^,它可能在人类神经退行性疾病的病因学和肿瘤发展中扮演重要角色^[29]^。CALM1在大多数的癌症(包括胰腺癌)中高表达,CALM1的表达还具有很高的诊断和预后潜力,可作为研究胰腺癌临床预后的候选标志物^[30]^, CALM1表达的升高有助于激活癌症相关通路,如WNT和MAPK通路,其表达在多种癌症中受DNA甲基化调控,与巨噬细胞和中性粒细胞的浸润水平呈显著正相关^[31]^。TCOF1在核糖体生物发生、DNA损伤应答(DDR)、有丝分裂调控和端粒完整性等多个过程中发挥着关键作用,TCOF1可能会影响ATPase活性、微管结合、微管蛋白结合和DNA催化活性,并通过调控“细胞周期”和“细胞衰老”途径参与肿瘤发生过程^[32]^,肿瘤组织的TCOF1表达水平高于正常组织,可影响癌症的预后,并与免疫细胞浸润相关^[33]^。

综上,胰腺癌患者样本中11个上调的差异表达蛋白质与肿瘤的发生发展密切相关,其中EXTL2、A2ML1和PARK7的异常表达最为显著,这些蛋白质为胰腺癌的诊断和治疗预后提供了重要的参考价值,也表明所开发的磷脂分子印迹材料在血浆外泌体蛋白质组领域具有潜在的应用价值,可为临床诊断和治疗提供更好的分子工具与解决方案。

## 3 结论

本研究将PS-MIP用于血浆样本的外泌体富集和多种表征,外泌体标志蛋白CD9、TSG101和CD81的免疫印迹分析表明了富集的效率和特异性,TEM和NTA的形貌和粒径分布显示富集的外泌体具有完整性。将其应用于胰腺癌患者血浆外泌体的富集和蛋白质组分析,筛选到表达上调的蛋白质11个和下调的蛋白质24个,通过PPI网络分析,连接度相对较高的前3个基因是补体因子D基因(complement factor D,*CFD*)、补体C3基因(complement component 3,*C3*)、血管性血友病因子基因(von willebrand factor,*VWF*);在胰腺癌患者外泌体的蛋白质组学表达上调的蛋白质中,EXTL2和A2ML1、PARK7的过度表达最为显著,这些蛋白质可能是胰腺癌诊断和预后评估的生物标志物,为基于外泌体的肿瘤液体活检提供了重要的科学参考。

## References

[1] Pegtel D M, Gould S J. Annu Rev Biochem, 2019, 88: 487 31220978 10.1146/annurev-biochem-013118-111902

[2] Kalluri R, LeBleu V S. Science, 2020, 367(6478): eaau6977 10.1126/science.aau6977 PMC7717626 32029601

[3] Weng Y J, Sui Z G, Zhang L H, et al. Chinese Journal of Chromatography, 2016, 34(12): 1131

[4] LeBleu V S, Kalluri R. Trends Cancer, 2020, 6(9): 767 32307267 10.1016/j.trecan.2020.03.007

[5] Yang K G, Wang W W, Wang Y, et al. Chinese Journal of Chromatography, 2021, 39(11): 1191 34677014 10.3724/SP.J.1123.2021.04009 PMC9404187

[6] Livshts M A, Khomyakova E, Evtushenko E G, et al. Sci Rep, 2015, 5: 17319 26616523 10.1038/srep17319 PMC4663484

[7] Gao F Y, Jiao F L, Zhang Y J, et al. Chinese Journal of Chromatography, 2019, 37(10): 1071 31642286 10.3724/SP.J.1123.2019.02007

[8] Zhou J T, Cheng X H, Guo Z C, et al. Angew Chem Int Ed Engl, 2023, 62(19): e202213938 36916765 10.1002/anie.202213938

[9] Klein A P. Nat Rev Gastroenterol Hepatol, 2021, 18(7): 493 34002083 10.1038/s41575-021-00457-x PMC9265847

[10] Park W, Chawla A, O'Reilly E M. JAMA, 2021, 326(9): 851 34547082 10.1001/jama.2021.13027 PMC9363152

[11] Hayashi A, Hong J, Iacobuzio-Donahue C A. Nat Rev Gastroenterol Hepatol, 2021, 18(7): 469 34089011 10.1038/s41575-021-00463-z

[12] Kim J E, Lee K T, Lee J K, et al. J Gastroenterol Hepatol, 2004, 19(2): 182 14731128 10.1111/j.1440-1746.2004.03219.x

[13] Huang P, Gao W, Fu C, et al. Mol Cell Proteomics, 2023, 22(7): 100575 37209817 10.1016/j.mcpro.2023.100575 PMC10388587

[14] Kong A T, Leprevost F V, Avtonomov D M, et al. Nat Methods, 2017, 14(5): 513 28394336 10.1038/nmeth.4256 PMC5409104

[15] Miyanishi M, Tada K, Koike M, et al. Nature, 2007, 450(7168): 435 17960135 10.1038/nature06307

[16] Nadanaka S, Kitagawa H. Matrix Biol, 2014, 35: 18 24176719 10.1016/j.matbio.2013.10.010

[17] Zhang W M, Cui Q C, Qu W F, et al. Oncol Rep, 2018, 40(1): 206 29749538 10.3892/or.2018.6426 PMC6059744

[18] Kong L M, Liu P, Zheng M J, et al. Epigenomics, 2020, 12(6): 507 32048534 10.2217/epi-2019-0374

[19] He X, Zheng Z, Li J, et al. Carcinogenesis, 2012, 33(3): 555 22223849 10.1093/carcin/bgs002

[20] Inberg A, Linial M. J Biol Chem, 2010, 285(33): 25686 20516060 10.1074/jbc.M110.109751 PMC2919132

[21] Zhou X, Zhu X, Yao J, et al. Invest New Drugs, 2021, 39(6): 1469 33983530 10.1007/s10637-021-01128-z

[22] Scielzo C, Bertilaccio M T, Simonetti G, et al. Blood, 2010, 116(18): 3537 20530793 10.1182/blood-2009-12-258814

[23] Liao X, Huang K, Huang R, et al. OncoTargets Ther, 2017, 10: 4493 10.2147/OTT.S142557 PMC5602474 28979141

[24] Chang T Y, Li B L, Chang C C, et al. Am J Physiol-Endocrinol Metab, 2009, 297(1): E1 10.1152/ajpendo.90926.2008 PMC2711667 19141679

[25] Li J, Gu D, Lee S S Y, et al. Oncogene, 2016, 35(50): 6378 27132508 10.1038/onc.2016.168 PMC5093084

[26] Long J, Luo G, Liu C, et al. Int J Oncol, 2012, 41(5): 1662 22941445 10.3892/ijo.2012.1613

[27] Yokoi K, Shih L C N, Kobayashi R, et al. Int J Oncol, 2005, 27(5): 1361 16211233

[28] Terenzio M, Di Pizio A, Rishal I, et al. Neurobiol Dis, 2020, 140: 104816 32088381 10.1016/j.nbd.2020.104816 PMC7273200

[29] Jiang J M, Yu L, Huang X H, et al. Gene, 2001, 281(1/2): 103 11750132 10.1016/s0378-1119(01)00787-9

[30] Lei Y Y, Yu T Z, Li C Y, et al. J Cell Mol Med, 2021, 25(2): 1198 33342045 10.1111/jcmm.16188 PMC7812292

[31] Yao M L, Fu L Y, Liu X D, et al. Front Genet, 2022, 12: 793508 35096010 10.3389/fgene.2021.793508 PMC8790318

[32] Gu W, Sun L, Wang J, et al. Aging-US, 2022, 14(2): 943 10.18632/aging.203852 PMC8833134 35093935

[33] Hu J Y, Lai Y N, Huang H, et al. Br J Cancer, 2022, 126(1): 57 34718356 10.1038/s41416-021-01596-3 PMC8727631

